# Bioprospecting for Novel Probiotic Strains from Human Milk and Infants: Molecular, Biochemical, and Ultrastructural Evidence

**DOI:** 10.3390/biology11101405

**Published:** 2022-09-26

**Authors:** Sabry Y. M. Mahmoud, Atallah A. Atallah, Omnia A. Badr, Mahmoud M. A. Moustafa, Ahmed Esmael, Nesrine Ebrahim, Mohammed Aljeldah, Basim Al Shammari, Ibrahim A. Alsafari, Shereen A. Mohamed

**Affiliations:** 1 Biology Department, College of Sciences, University of Hafr Al Batin, P.O. Box 1803, Hafar Al-Batin 31991, Saudi Arabia; 2 Department of Dairy Science, Faculty of Agriculture, Benha University, Moshtohor 13736, Egypt; 3 Genetics and Genetic Engineering Department, Faculty of Agriculture, Benha University, Moshtohor 13736, Egypt; 4 Botany and Microbiology Department, Faculty of Science, Benha University, Benha 13518, Egypt; 5 Department of Medical Histology and Cell Biology, Faculty of Medicine, Benha University, Benha 13511, Egypt; 6 Department of Clinical Laboratory Sciences, College of Applied Medical Science, University of Hafr Al Batin, P.O. Box 1803, Hafar Al Batin 31991, Saudi Arabia

**Keywords:** human milk, probiotic bacteria, antibacterial activity, 16S rRNA gene

## Abstract

**Simple Summary:**

Demographic, genetic factors, and maternal lifestyle could modify and alter the microbial diversity of human milk and infants’ gut. We screened human breast milk and infant stool samples from Egyptian sources for possible novel probiotic strains. Forty-one isolates were submitted to the gene bank database, classified, and identified through physiological and biochemical tests. All samples revealed antibiotic resistance, antibacterial activity, and high probiotic features. Six of the isolates revealed less than 95% Average Nucleotide Identity with deposited sequences in the database. Isolate *Lactobacillus delbrueckii* ASO 100 exhibited the lowest identity ratio with promising probiotic and antibacterial features, enlightening the high probability of being a new probiotic species.

**Abstract:**

Human milk comprises a diverse array of microbial communities with health-promoting effects, including colonization and development of the infant’s gut. In this study, we characterized the bacterial communities in the Egyptian mother–infant pairs during the first year of life under normal breastfeeding conditions. Out of one hundred isolates, forty-one were chosen for their potential probiotic properties. The selected isolates were profiled in terms of morphological and biochemical properties. The taxonomic evidence of these isolates was investigated based on 16S rRNA gene sequence and phylogenetic trees between the isolates’ sequence and the nearest sequences in the database. The taxonomic and biochemical evidence displayed that the isolates were encompassed in three genera: *Lactobacillus*, *Enterococcus*, and *Lactococcus*. The *Lactobacillus* was the most common genus in human milk and feces samples with a high incidence of its different species (*Lacticaseibacillus paracasei*, *Lactobacillus delbrueckii*, *Lactiplantibacillus plantarum*, *Lactobacillus gasseri*, and *Lacticaseibacillus casei*). Interestingly, BlastN and Jalview alignment results evidenced a low identity ratio of six isolates (less than 95%) with database sequences. This divergence was supported by the unique physiological, biochemical, and probiotic features of these isolates. The isolate *L. delbrueckii*, ASO 100 exhibited the lowest identity ratio with brilliant probiotic and antibacterial features suggesting the high probability of being a new species. Nine isolates were chosen and subjected to probiotic tests and ultrastructural analysis; these isolates exhibited antibiotic resistance and antibacterial activity with high probiotic characteristics, and high potentiality to be used as prophylactic and therapeutic agents in controlling intestinal pathogens.

## 1. Introduction

The American Academy of Pediatrics evidenced the prophylactic and therapeutic roles of human milk in educating infants’ immune systems and providing protection against many infectious diseases such as gastrointestinal, respiratory, inflammatory bowel, and allergic diseases [1,2]. These protective effects of breast milk are due to the orchestrated action of several bioactive molecules, such as oligosaccharides, fatty acids, immunoglobulins, cytokines, immune cells, lactoferrin, immunomodulating factors, and healthy microbial communities [3].

Breast milk is the second integral source of infant microbes after the birth canal in vaginally born infants [4]. It has been predicted that an infant takes approximately 10^5^–10^7^ commensal bacteria every day via consuming 800 mL of breast milk. Human breast milk contributes a distinctive role in the initiation, development, and composition of the neonatal gut microbiota. The human milk microbiome has a diverse array of bacterial species, including beneficial, commensal, and potentially probiotic bacteria [5].

Probiotics are live bacteria that deliver health benefits to the host when consumed at adequate levels as described by the Food and Agriculture Organization (FAO) and the World Health Organization (WHO) [6]. Probiotic bacteria have many beneficial characteristics such as the ability to colonize and dominate in the neonatal gut, ability to resist stomach acid and bile salts, adherence to the intestinal mucosa, initiation of anti-inflammatory responses, inhibition of pathogens by the production of antimicrobial constituents, and augmentation of the immune system [7,8,9].

A new era of therapeutics is in perspective in which probiotics and their purified molecules will be employed as a wise, safe alternative to medication and other treatments to control health imbalance and diseases in humans and animals [10]. In this manner, several studies have reported the role of probiotics in the prevention and treatment of inflammatory bowel disease [11,12] food hypersensitivity [13], cardiometabolic disorders [14], and antitumor activity [15]. 

Previous studies have evidenced the richness of human milk and feces samples with promising novel probiotics. The investigation by Lee et al. [16] reported the presence of novel *Lactobacillus gasseri* EJL and *Bifidobacterium breve* JTL strains in the milk and feces samples of Korean Mother-infant pairs. A recent interesting study by Li et al. [17] identified novel LAB bacterial strains belonging to *Lactobacillus gasseri*, *Lactiplantibacillus plantarum*, and *Lacticaseibacillus rhamnosus* from Chinese infants with potential probiotic characteristics against inflammation and oxidative stress-related human diseases.

The microbial diversity of human milk and consequently, infants’ gut is controlled by environmental, demographic and genetic factors, and the maternal lifestyle. Bioprospecting the gut microbiota, and selecting promising probiotic candidates, is of great importance to the new insights of personalized medicine. In the current investigation into future perspectives in treating chronic diseases, we prospect for such healthy probiotics from Egyptian populations characterized by unique immune systems. Targeted bacterial isolates were profiled in terms of morphological, biochemical, and ultrastructural properties. Probiotic tests, antibiotic susceptibility, and antibacterial activity were also considered. The taxonomic evidence of these isolates was demonstrated based on 16S rRNA gene sequence and phylogenetic tree analysis. 

## 2. Materials and Methods

### 2.1. Isolation, Phenotypic and Biochemical Features of Lactic Acid Bacteria

We recruited healthy mothers and their infants as volunteers from the community of the Faculty of Agriculture, Benha University, Moshtohor, Qalyubia, Egypt. Breast milk and stool samples were collected from 18 mother-baby pairs. Fresh feces from healthy infants between one and twelve months of age were collected during home study visits. Breast milk samples were collected from mothers (25–35 years) in sterile tubes using a manual expression with sterile gloves after cleaning the nipples and areola by wiping with a swab soaked in sterile water.

Ten grams from each fecal sample were diluted in 90 mL of sterile peptone water (0.1 g/L, Merck, Darmstadt, Germany). One ml of fresh breast milk was diluted in sterile peptone water. A series of dilutions of the fecal and milk samples were performed in peptone water, and bacteria in those samples were cultured by deploying the pour plate method on either MRS (pH 6.4) or MRS-cysteine agar (pH 5.5, 0.05%), and M17 agar media to selectively isolate the presumptive lactic acid bacteria (LAB). Plates were incubated at 37 °C for 72 h under anaerobic conditions (in an anaerobe jar using Oxoid AnaeroGen Compact). Single pure colonies were picked up and purified through three successive subcultures on the MRS medium. All the purified isolates were preserved in MRS broth containing 20% (*v*/*v*) glycerol as frozen stocks at −40 °C.

Isolated pure cultures were identified as LAB by cell morphology, Gram staining, catalase, and oxidase reaction [18,19,20]. In addition, LAB isolates were tested for gas production from glucose in MRS broth with an inverted Durham tube [21]. The Carbohydrate fermentative profile of the LAB was investigated against a cohort of 17 different sugars [21]. Further biochemical tests were performed to confirm the presumptive LAB isolates. Isolates that showed Gram-positive, catalase, and oxidase negative were selected as presumptive LAB and were further confirmed using the 16S rDNA genome typing.

### 2.2. Molecular Identification of LAB Isolates by 16S rRNA Sequencing

#### 2.2.1. Genomic DNA Extraction

Genomic DNA was extracted from fresh lactic acid bacterial isolates using QIAamp DNA Micro Kit, Cat. No./ID: 56304, following the manufacturer’s instructions. The concentration and purity of purified DNA were assessed on a BioTek Epoch 2 spectrophotometer (Thermo Scientific, Waltham, MA, USA). DNA integrity was checked on 1% agarose gel electrophoresis followed by visualization using a gel Doc™ EZ imaging system with image lab™ software (Bio-Rad, Hercules, CA, USA).

#### 2.2.2. Amplification of 16S rDNA of Isolates

Universal 16S rRNA primers 27F: 5′-AGAGTTTGGATCMTGGCTCAG-3′ and 1492R: 5′-CGGTTACCTTGTTACGACTT-3′ were utilized for DNA amplification [22,23]. PCR reaction volume of 50 µL contained 0.4 μM of each primer with a concentration of 10 pM, 400 μM of dNTP mix, 5 µL PCR reaction buffer (10×), 2 μM MgCl_2_, 2.5 units of TAKARA Taq DNA polymerase, 1 μL of template DNA, and the final volume was adjusted with sterilized double water. The PCR program was performed by applying a thermal cycle PCR machine (SensoQuest, Göttingen, Germany) as follows: initial denaturation at 95 °C for 3 min; then 35 cycles of denaturation at 95 °C for 50 s, annealing at 55 °C for 1 min, and extension at 72 °C for 1 min; followed by a final extension at 72 °C for 10 min. Amplified PCR fragments were subjected to 2% agarose gel electrophoresis and stained with ethidium bromide using a GeneRuler™ 1 kb DNA ladder, followed by visualization using a gel Doc™ EZ Imaging System with Image Lab™ Software (Bio-Rad, Hercules, CA, USA).

#### 2.2.3. Sequencing and Phylogenetic Analysis

PCR amplicons were purified according to the instructions of QIAquick PCR Purification Kit. Purified amplicons were sequenced in Macrogen Company in South Korea. To determine closely related bacteria, the 16S rRNA sequences were aligned and compared with known sequences in the NCBI nucleotide database using the BLASTn algorithm. Jalview software [24] (http://www.jalview.org/ accessed on 12 September 2021) was utilized to detect Single-nucleotide polymorphisms (SNPs) and pairwise sequence alignment between each acquired sequence (isolate) and the nearest deposited sequences in the NCBI database. Phylogenetic tree construction was performed to evidence the evolutionary relationship among the isolates and the closest ones in the database using the Maximum Likelihood method based on the Tamura-Nei model with MEGA X software [25]. 

### 2.3. Scanning Electron Microscopy 

Based on the previous morphological, biochemical, and physiological characterization of the isolates and molecular evidence of the 16S rRNA sequences, nine unconventional representative isolates from different species were chosen and subjected to ultrastructural analysis and probiotic tests.

#### 2.3.1. Growth Conditions

All the strains were in the form of pure frozen cultures and were sub-cultured three times in growth media containing 10% skimmed milk powder (*w*/*v*; Merck, Darmstadt, Germany) supplemented with 0.2% yeast extract (*w*/*v*; Difco, Beirut, Lebanon). The growth temperature was 37 °C for all strains except those of the cocci which were incubated at 30 °C, the inoculum amount was 3% for the bacilli strains and 2% for all the others. The incubation time was 6 h for the cocci strains and 8 h for the bacilli strains.

#### 2.3.2. Isolate Preparation for SEM

After the third subculture, 0.5 mL were taken from the coagulated media and washed with 0.5 mL of phosphate buffer (Merck, 0.1 mol L^−1^; pH 7.2). After three washes, each was followed by centrifugation at 2000 rpm for 10 min; the cells were resuspended in 1 mL of the buffer [26]. A small number of cells (approximately 200 µL) were attached to poly-L-lysine coated cover glass and fixed in 2% (*v*/*v*; Merck) Glutaraldehyde. Cells were rinsed with 0.1 M sodium cacodylate (Merck; pH 7.4) buffer, post-fixed in 1% OsO_4_ (Merck), and exposed to thio-carbohydrazide (Merck) as described by [27]. Each sample was fixed on an iron stub and then made electrically conductive by coating it (in a vacuum chamber) with a thin layer of gold for 40 s. The moisture of freeze-dried samples was completely removed by placing the freeze-dried sample in an air-tight desiccator containing silica gel. The weight of samples was periodically measured until constant weight to confirm the complete removal of moisture. At least four images of typical structures at 1500 magnification were recorded using a scanning electron microscope (FEI Company, Eindhoven, The Netherlands) model quanta 250 FEG (field emission gun) attached with EDX unit (energy dispersive x-ray analyses), The images were taken at an excitation voltage of 20 K.V., at different magnifications varying from 400 to 6000 and working distance varying from 13.7–14.2 mm. Only 5000 magnification was shown for the present study.

### 2.4. Probiotic Characteristics of Isolates

#### 2.4.1. Acidity Resistance

LAB isolates (1 mL of each isolate) were inoculated individually into MRS and M17 broth (10 mL). MRS broth was adjusted to pH 3 and M17 broth was adjusted to pH 6.4 and incubated at 37 °C for 1, 2, and 3 h. Viable counts of the acid-tolerant bacteria were enumerated after incubation aerobically or anaerobically at 37 °C for 48 h [28].

#### 2.4.2. Bile Salt Tolerance

Ox-gall salt media was applied to study the bile tolerance of the LAB isolates [28]. One ml of Activated isolates was inoculated into 10 ml MRS and M17 broth media containing 0.5% of the ox-bile salt. The control comprised MRS and M17 broth without bile salt. The viable bacteria were enumerated after incubation aerobically or anaerobically at 37 °C for 48 h.

#### 2.4.3. Bile Salt Hydrolase Activity Assay

The isolates were tested for bile salt hydrolase on MRS and M17 agar fortified with 0.5% sodium salts of tauro-deoxycholic acid (TDCA) [29]. Activated isolates were inoculated and plated onto MRS and M17 agar containing TDCA. The plates were incubated anaerobically or aerobically at 37 °C for 48 h. Bile salt hydrolase activity was indicated by deoxycholic acid precipitate around the colonies.

#### 2.4.4. Antagonistic Activity

The antagonistic activity of the LAB isolates against three pathogenic bacteria was carried out by the agar diffusion test. The targeted pathogens were activated in tryptic soy broth (TSB). one hundred microliter of the test bacteria were spread onto Muller-Hinton agar plates. Plates were air dried for 15 minutes and discs were impregnated with 30 µL of cell-free filtered supernatants (obtained by centrifugation of the LAB cultures at 5000 rpm for 5 min). The plates were incubated at 37 °C for 24 h, and the diameter of inhibition zones (mm) was measured around the discs [30]. The experiment was performed in triplicate for each LAB isolate. The antagonistic activity was tested against *Bacillus subtilis*, *Staphylococcus aureus*, and *Escherichia coli*.

#### 2.4.5. Antibiotic Susceptibility Testing

Antibiotic resistance of the isolates was tested against five selected antibiotics (Oxoid, UK); Tetracycline (30 μg), Neomycin (30 μg), Vancomycin (30 μg), Kanamycin (30 μg), and Streptomycin (10 μg). Isolates were grown in MRS and M17 broth at 37 °C for 24 h. Overnight isolates were inoculated into MRS and M17 broth and freshly diluted 1: 10 in MRS and M17 broth; 0.1 mL of each diluted isolate was inoculated into MRS and M17 agar kept at 45 °C and poured into Petri plates to solidify. After solidification of the inoculated agar plates, antibiotic discs were placed on the surface of the plates. After 24 h incubation at 37 °C, the diameters of inhibition zones around the discs were measured (mm) according to [31]. Data (average of two determinations) were expressed in terms of resistance (R); moderate susceptibility (MS) and susceptibility (S); according to the guidelines of the Clinical and Laboratory Standards Institute (CLSI) [32].

#### 2.4.6. Statistical Analysis

All values were expressed as a mean of six replicates ± SE. Differences between isolates were estimated by one-way analysis of variance using SAS software [33]. Differences were significant at *p* ≤ 0.05. A Duncan multiple ranges test [34] was utilized to evaluate the significant differences among means.

## 3. Results

### 3.1. Isolation and Identification of Lactic Acid Bacteria

Out of 100 bacterial isolates, only 41 isolates were chosen as gram-positive, catalase-negative, and non-endospore forming lactic acid bacterial isolates as shown in Table 1. All the cocci isolates were positive for facultative anaerobic or microaerophilic tests. Under microscopic investigation, only 14 isolates were identified as cocci-shaped bacteria and 39 were characterised as rod-shaped bacteria.

### 3.2. Physiological and Biochemical Characteristics

Physiological and biochemical characteristics for Cocci isolates are presented in Table 2. All Cocci isolates were resistant to growth at 4.0% bile salt and pH 6.8. On another hand, they revealed a negative profile for growth at pH 9.6 and gas production ability. For Growth at different temperatures, they have grown successfully at 40 °C, 10 °C, and negatively at 45 °C. All isolates showed positive results for growth at different NaCl levels (4 and 6.5%), except ASO26 and ASO290 isolates, which were negative for growth at 6.5% NaCl. Positive abilities to hydrolyse arginine, coagulate milk and produce acid from glucose were detected in all studied isolates. Sugar fermentation profiles and the ability to produce acid from lactose, raffinose, salicin, fructose, glucose, and mannose were positive for all strains. However, these isolates displayed a negative ability to produce acid from mannitol, ribose, trehalose, sorbitol, and xylose. 

As shown in Table 3, the 25-rod lactic acid bacterial isolates revealed a positive ability to grow at 4.0% bile salt, pH 6.8, and pH 9.6 with a negative ability for gas production. For Growth at different temperatures, they have grown successfully at 45 °C and negatively at 15 °C. All isolates showed positive results for growth at 6.5% NaCl levels. Positive abilities to hydrolyse arginine and coagulate milk were detected in all studied isolates. Sugar fermentation profiles and the ability to produce acid from lactose, galactose, glucose, fructose, maltose, sucrose, mannose, rhamnose, arabinose, and melibiose were positive for all strains. However, these isolates displayed a negative ability to produce acid from ribose, mannitol, ribose, salicin, and sorbitol. Interestingly, all isolates displayed positive profiles for acid production from xylose, raffinose, and trehalose except ASO57, ASO55, ASO5, and ASO100 which showed a negative profile for xylose, raffinose, and trehalose. 

### 3.3. Molecular Identification

The amplified PCR products of the 16S rRNA gene of the selected 41 isolates were sequenced and deposited in the NCBI database. The accession numbers of the isolates and their identity ratio with the closest deposited sequences in the database are shown in Table 4. The Pairwise sequence alignment results revealed the presence of three genera, *Lactobacillus*, *Enterococcus* and *Lactococcus*. A high incidence of *Lactobacillus* species (*L. paracasei*, *L. delbrueckii*, *L. plantarum*, *L. gasseri* and *L. casei*) was found in human milk and feces. Fifteen different isolates belonged to *L. paracasei* and four isolates belonged to *L. delbrueckii*. However, only two isolates were detected for each species of *L. plantarum* and *L. gasseri* and one isolate belonged to *L. casei*. In addition, the genus *Enterococcus* revealed a high incidence with twelve isolates scattered in the species *E. faecium*, *E. faecalis*, and *E. lactis*.

Interestingly, BlastN and Jalview alignment results of the six isolates evidenced a low identity ratio ranging from 81.39% to 94.63% for the accession numbers of MZ930471, OK030542, OK033573, OK021544, OK032511, and OK032119. Moreover, 11 isolates showed identity from 95% to 97%, 14 isolates displayed identity from 97% to 98% and only 10 isolates were from 98.14% to 98.95% identity ratio in comparison with the closest similar sequences in the database.

**Figure 1:**
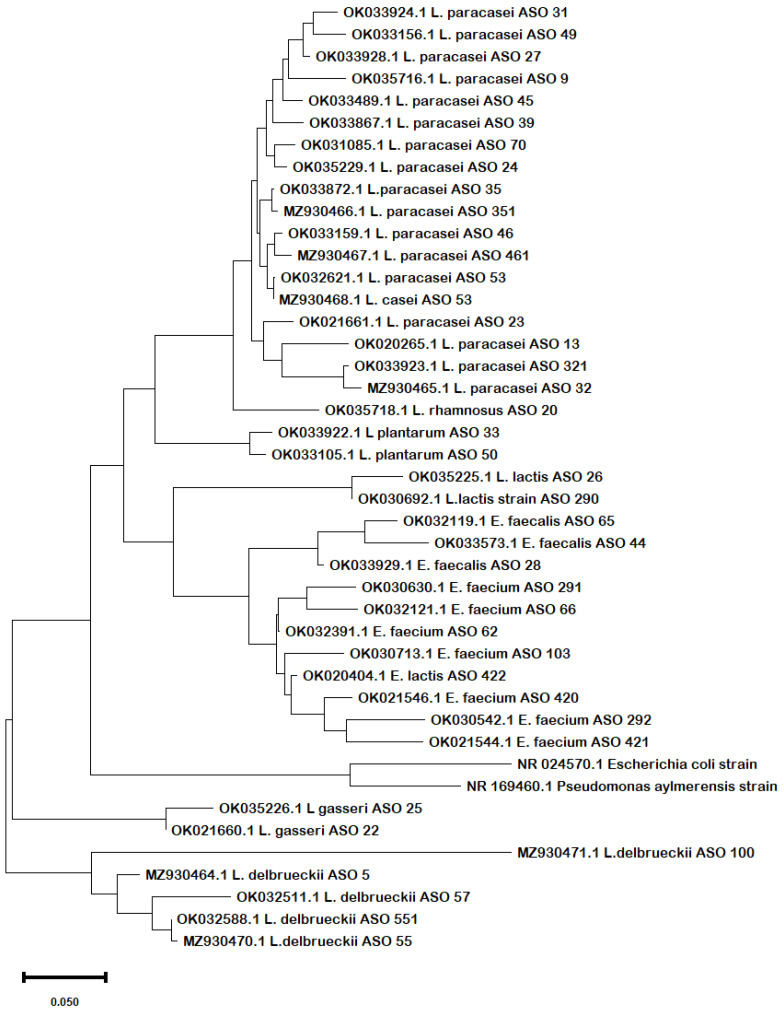
Phylogenetic tree shows the evolutionary relationships between the 16S rRNA sequences of the obtained concatenated nucleotide sequences of their 16S rRNA. The Maximum Likelihood tree was constructed using the MEGA X software with the Maximum Likelihood algorithm and default setting. The bar length represents 0.05 substitutions per nucleotide site. Branch support was estimated from 1000 bootstrap replicates. *E. coli* and *Pseudomonas* 16S rRNA sequences serve as outgroups to root the tree.

Phylogenetic analysis of the obtained sequences confirmed the same results in terms of the evolutionary relationship among the strains. From the phylogenetic tree (Figure 1), it can be inferred that there was a clear similarity among the different species of *Lactobacillus*, *Lacticaseibacillus*, and *Lactococcus* except for *L. delbrueckii* and *L. gasseri* that diverged in different clades. The species of *Enterococcus* spp. were clustered together, revealing close similarities.

### 3.4. Scanning Electron Microscopy (SEM)

**Figure 2:**
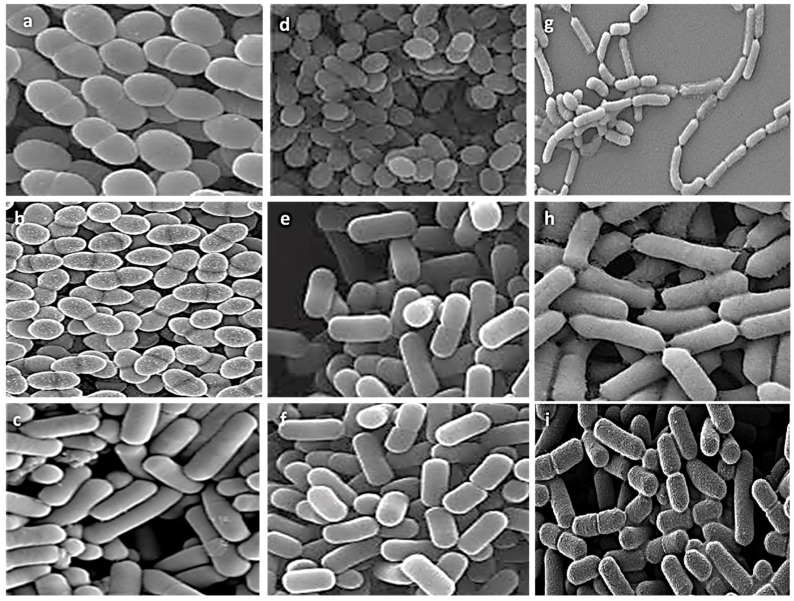
Scanning electron microscopy visualization of different isolates, (**a**) *E. faecalis* ASO44; (**b**) *E. faecium* ASO292; (**c**) *L. delbrueckii* ASO100; (**d**) *L. lactis* ASO26; (**e**) *L. plantarum* ASO50; (**f**) *L. case* ASO53; (**g**) *L. rhamnosus* ASO20; (**h**) *L. gasseri* ASO25 (**i**) *L. paracasei* ASO32. Images were captured at an excitation voltage of 20 K.V., at 5000 magnification with a working distance of 13.7–14.2 mm.

Nine representative isolates from different genera were selected for further experiments based on literature evidence of their probiotic characteristics and the uniqueness of their biochemical, physiological, and 16S rRNA molecular profiles. Morphological ultrastructure features of these isolates are presented in Figure 2. Images of the isolates showed the presence of three cocci isolates (*E. faecalis* ASO44, *E. faecium* ASO292, *L. lactis* ASO26) and six bacilli isolates (*L. delbrueckii* ASO100, *L. plantarum* ASO50, *L. casei* ASO53, *L. rhamnosus* ASO20, *L. gasseri* ASO25, *L. paracasei* ASO32). There were obvious differences in cell shapes and assemblies of all investigated isolates.

### 3.5. Probiotic Characteristics

**Figure 3:**
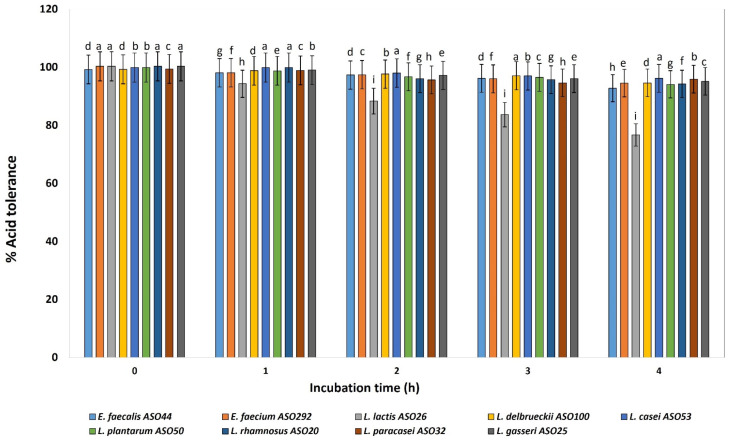
Percentage of acid tolerance of the isolated lactic acid bacteria during different incubation times of 0, 1, 2, 3 and 4 h. LAB isolates were grown on pH 3.0 for 4 h, and percentages of tolerance were estimated by counting viable bacterial counts which tolerated the acidic medium with relative to neutral pH conditions (pH 6.4). ^a–i^ Estimates with the same letters are not significantly different (*p* > 0.05) among different isolates for the same incubation time.

Nine isolates from different species were selected for focusing on their probiotic and ultrastructural characteristics. All investigated isolates revealed acid tolerance and survived very well and were not affected by decreasing the pH value from 6 to 3 for 4 h after incubation (Figure 3). The viable cell count of all isolates remained higher than 6–7 Log CFU mL^−1^. At pH 3, slight decreases were determined in the cell count of *L. lactis* ASO 26. Relatively log CFU mL^−1^ increases were determined for *L. delbrueckii* ASO100, *L. paracasei* ASO32, and *L. plantarum* ASO50, for 4 h at pH 3. According to this test, all isolates were resistant to low pH except *L. lactis* ASO 26 which was sensitive to low pH. 

**Figure 4:**
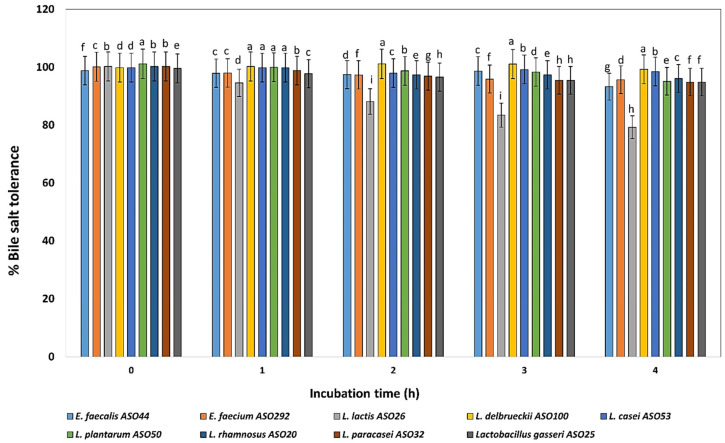
Percentage of bile tolerance of the isolated lactic acid bacteria during different incubation times of 0, 1, 2, 3, and 4 h. LAB isolates were grown on 0.5% ox-bile salt for 4 h, and percentages of tolerance were estimated by counting viable bacterial counts which tolerated the bile salt with relative to untreated controls (with no bile salts). ^a–i^ Estimates with the same letters are not significantly different (*p* > 0.05) among different isolates for the same incubation time.

The bile salt (ox-gall bile salts 0.5%) tolerance test revealed that all isolates grew well in the presence of 0.5 % bile salts during incubation at 4 h except, *L. lactis* ASO 26 which showed declined profile during the incubation time. On another hand, *L. delbrueckii* ASO100, *L. paracasei* ASO32, and *L. plantarum* ASO50 exhibited the highest bile salts tolerance, respectively (Figure 4). 

**Figure 5:**
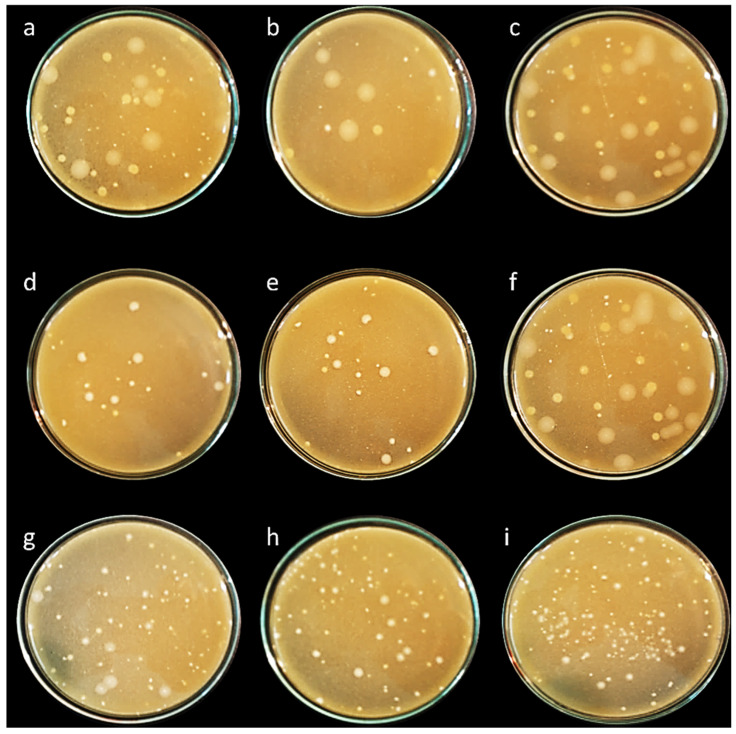
Bile salt hydrolase’s activity assay of isolated lactic acid bacteria. Activated LAB isolates were inoculated and plated onto MRS and M17 agar containing tauro-deoxycholic acid. The plates were incubated anaerobically or aerobically at 37 °C for 48 h. Bile salt hydrolase activity was indicated by deoxycholic acid precipitate around the colonies. (**a**) *E. faecalis* ASO44; (**b**) *E. faecium* ASO292; (**c**) *L. delbrueckii* ASO100; (**d**) *L. lactis* ASO26; (**e**) *L. plantarum* ASO50; (**f**) *L. casei* ASO53; (**g**) *L. rhamnosus* ASO20; (**h**) *L. gasseri* ASO25 (**i**) *L. paracasei* ASO3.

All isolates expressed bile salts hydrolase activity and deconjugated ability with taurine or glycine-bile acid or both. This activity showed as a hole around the colonies after growth in agar plate supplemented with 0.5% TDCA or 0.5% GDCA. The isolates *L. delbrueckii* ASO100 and *L. casei* ASO53 exhibited the highest bile salts hydrolase activity (Figure 5).

Regarding antibiotic resistance, all studied isolates were resistant to kanamycin, tetracycline, neomycin, streptomycin, and vancomycin except, *L. lactis* ASO26, which was sensitive to streptomycin and vancomycin. The isolates *L. delbrueckii* ASO100 and *L. casei* ASO53 displayed the highest resistance pattern against kanamycin, tetracycline, neomycin, and streptomycin. However, the isolate *L. rhamnosus* ASO20 revealed a high resistant profile against vancomycin (Table 5).

**Figure 6:**
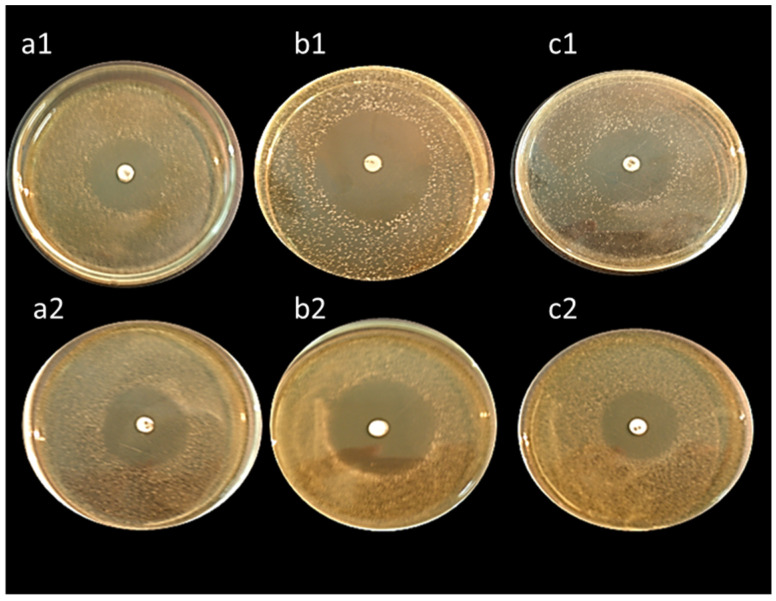
Antibacterial activity of cell-free supernatants of *L. delbrueckii* ASO100 (upper three plates) and *L. rhamnosus* ASO20 (lower three plates) against *Bacillus subtilis* (**a1**,**a2**), *Staphylococcus aureus* (**b1**,**b2**), and *Escherichia coli* (**c1**,**c2**). The plates were firstly inoculated with the tested pathogenic bacteria, then allowed to air dry for 15 min; discs impregnated with cell-free supernatants were spotted on the middle of the plates. Diameters of the formed inhibition zones were measured after incubation at 37 °C for 24 h.

All isolates exhibited high antibacterial activity against *Bacillus subtilis*, *Staphylococcus aureus,* and *Escherichia coli* except, *L. lactis* ASO26 which did not reveal any antibacterial activity (Table 6). Interestingly, the isolates *L. delbrueckii* ASO100 and *L. rhamnosus* ASO20 displayed the highest antibacterial activity (Figure 6). 

## 4. Discussion

In this investigation, we seek new potent probiotics from the breast milk of Egyptian mothers and stool samples of their infants. The novel aspects of our study include (1) exploring human milk microbiota diversity from Egyptian samples for the first time and (2) isolating innovative probiotics from Egyptian infants’ feces that are characterized by a unique immune system as prophylactic and therapeutic agents for controlling chronic diseases.

Where lactic acid bacteria colonize gut epithelial cells and withstand pathogens and reactive oxygen species (ROS) associated with gut diseases, it should have the ability to endure harsh conditions in the human body (intestinal juice, low pH, and salivary enzymes) to maintain gut microbiota balance, immune homeostasis and monitor beneficial physiological roles in human health [33,34]. In this manner and based on morphological and physiological characteristics, we selected only gram-positive, catalase-negative, positive microaerophilic and non-endospore forming lactic acid bacterial isolates to be investigated in the current study. Rod isolates revealed a positive ability to grow at 4.0% bile salt, pH 6.8, pH 9.6, 6.5% NaCl, at 45 °C with positive abilities to hydrolyze arginine and coagulate milk in accordance with the investigations of Soni et al. [35] and Lackey et al. [36]. All isolates exhibited high abilities to ferment various carbohydrates and produce acid from lactose, galactose, glucose, fructose, maltose, sucrose, mannose, rhamnose, arabinose, and melibiose. However, these isolates displayed a negative ability to produce acid from ribose, mannitol, ribose, salicin, and sorbitol. These results agreed with the recent report of Li et al. [17] who isolated 27 gram-positive and catalase-negative strains from healthy infant feces and evidenced their negative profile to mannitol and sorbitol. Interestingly, unlike all isolates, ASO57, ASO55, ASO5, and ASO100 showed negative profiles for producing acid from xylose, raffinose, and trehalose reflecting unique profiles and possible different probiotic characteristics.

Pairwise sequence alignment of 16S rRNA sequences revealed the presence of three genera, *Lactobacillus*, *Enterococcus*, and *Lactococcus*. *Lactobacillus* was the most common in human milk and feces samples with a high incidence of its different species (*L. paracasei*, *L. delbrueckii*, *L. plantarum*, *L. gasseri* and *L. casei*); these results were matched with the previous study of Zhang et al. [37] who reported a high incidence of *Lactobacillus* strains in feces samples of Chinese babies. Interestingly, six of our isolates evidenced low identity ratios ranging from 81.39% to 94.63% (less than 95%) with database sequences. The isolate that revealed the lowest identity ratio (*L. delbrueckii*, ASO 100) expressed the highest antibiotic resistance, antibacterial and probiotic activity. This isolate could be a new species as reported by Thompson et al. [38] and Badr et al. [39], and strains from different microbial species share less than 95% Average Nucleotide Identity (ANI). The low identity ratio of this isolate specifically was evidenced by chemical, physiological and probiotic features. Hence, this isolate and probably the other five isolates that shared less than 95% Average Nucleotide Identity could be a new probiotic species with novel and unique characteristics. These isolates showed the nearest similarity to *E. faecium* and *L. delbrueckii* strains. The previous investigation by Evivie et al. [40] confirmed the medicinal usage of *L. delbrueckii* isolates as a probiotic against foodborne pathogens. Moreover, recent studies have evidenced the role of *E. faecium* as a promising probiotic candidate for both human and animal use [41,42,43,44]. Despite 16S rRNA being a very conserved region, six of our isolates revealed huge divergence in that region and this could be explained by the effect of environmental, demographic, genetic factors, and the maternal lifestyle on modifying the microbial diversity of human milk and infants’ gut [45].

Previous investigations evidenced the role of current isolated strains as a promising candidate probiotic for medicinal and industrial usage for humans and animals. An interesting study by Salaris et al. [46] revealed that *L. paracasei* is a promising candidate probiotic that exhibits prophylactic potential effect against SARS-CoV-2 infection. Moreover, Otaka et al. [47] indicated that *L. paracasei* was useful to alleviate depressive symptoms, partly through its association with an abundance of actinobacteria in the gut microbiota. Another investigation by Guerra et al. [48] showed that lactobacilli isolates from newborn stools exhibited different probiotic properties such as gastrointestinal tolerance, antibiotic susceptibility, inhibition of pathogen biofilm formation, absence of alfa or gamma-blood hemolysis, and lysozyme sensibility. Besides, Hill et al.’s investigation [49] showed that the *L. casei* clusters have the potential to be used prophylactically or therapeutically in diseases related to a disturbance to the gut microbiota. Probiotics like *L. plantarum* are beneficial bacteria that stimulate the digestive system, fight pathogenic microbes, and help the human body to produce vitamins. Many people take *L. plantarum* probiotic pills to heal or prevent complaints, including seasonal allergies and irritable bowel syndrome [50].

For testing the competing probiotic characteristics of our isolates, we selected nine representative isolates from different genera for further experiments based on literature evidence of their probiotic characteristics and the uniqueness of their biochemical, physiological, and 16S rRNA molecular profiles. We utilized scanning electron microscopy investigation for deep visualization of morphological ultrastructure features of these isolates confirming clear differences in cell shapes and assemblies of all investigated isolates. From the selected isolates there were three cocci isolates (*E. faecalis* ASO44, *E. faecium* ASO292, *L. lactis* ASO26) and six bacilli isolates (*L. delbrueckii* ASO100, *L. plantarum* ASO50, *L. casei* ASO53, *L. rhamnosus* ASO20, *L. gasseri* ASO25, *L. paracasei* ASO32). All isolates revealed aggregation ability confirming their ability to colonize gut epithelial cells.

Owing to market competition and demand, probiotics must be able to endure challenging environments including the acidic environment of the gastrointestinal tract (GIT), intestinal bile salts, and digestive enzymes. Most exogenous microbes die when ingested into the GIT because of the very low pH of the secreted gastric juice (pH of 2.0). It is expected that probiotic strains should be able to adapt and tolerate the acidic nature of the GIT as they pass by to colonize the gut of their host [51,52]. Moreover, pH tolerance is important for the improvement of fermented foods like yogurt and cheese that affect strain sustainability due to their high acidity. Interestingly, all the investigated isolates in this study revealed acid tolerance of the isolated LAB to the pH value from 6 to 3 for 4 h after incubation. LAB are known for their capability to tolerate acidic pH [53]. Our result resembles preceding studies that reported the survival of LAB strains against simulated gastric juice with a pH of 2.0 [54,55].

One of the major requirements for probiotic selection is the ability to survive and grow in the GIT, so they should be able to tolerate the intestinal bile salt. Probiotic physiological alterations, including exopolysaccharide synthesis and carbohydrate fermentation, are related to the resistance to elevated bile salts [56]. The adaptation of probiotics to bile salts is also connected to the structure of membrane proteins and fatty acids as well as the prevention of pathogen adherence to human mucus [57,58]. To compete with pathogens when employed in functional foods, probiotic strains must possess resistance to bile salts. Tolerance of an average level of 0.3% of the bile salt has been estimated in many studies for potential probiotic LAB candidates [59]. Interestingly, our results estimated that, except for the *L. lactis* ASO 26, all the LAB isolates in this study exhibited tolerance to 0.5% bile salts for a 4 h incubation period.

Regarding antibiotic resistance in probiotics, it is considered a safety concern, as antibiotic resistance encoding genes could transfer among the microorganism community of the gut. The genomic context of the antibiotic resistance determinants of the current study’s probiotic strains is unknown but a future follow-up study will be performed to go through whole genome sequencing of these isolates to ensure that the antibiotic resistance determinants are not present as part of mobile genetic elements. Previous evidence by [60] reported the lack of cytochrome-mediated electron transport in Lactobacillus genera, and the presence of D-Ala-D-lactate in their peptidoglycan, hence, their resistance to different antibiotics including streptomycin, and vancomycin is considered to be intrinsic. Consequently, LAB probiotic strains can be used safely as pills alongside or after antibiotic treatment to restore the gut microbiota homeostasis [61]. In this case, antibiotic resistance possesses a strong advantage in order for probiotics to survive under antibiotic treatment conditions. In the current study, all studied isolates were resistant to kanamycin, tetracycline, neomycin, streptomycin, and vancomycin except, *L. lactis* ASO26, which was sensitive to streptomycin and vancomycin. The literature evidenced that most LAB species are resistant to kanamycin [62,63]. Remarkably, all isolates were resistant to streptomycin and vancomycin, in harmony with previous reports [64,65].

All tested isolates exhibited high antibacterial activity against *Bacillus subtilis*, *Staphylococcus aureus*, and *Escherichia coli* except, *L. lactis* ASO26 which did not reveal any antibacterial activity. Our results were in coincidence with Klayraung et al. [66], who investigated the antibacterial activity of lactobacilli isolated from four kinds of traditional fermented foods on *Staphylococcus aureus*, *Salmonella typhi*, and *Escherichia coli*, reporting the high antibacterial potency of LAB against *S. aureus*, *S. typhi*, and *E. coli*. The antibacterial activity of lactic acid bacteria could be explained by their production of a wide variety of different inhibitory substances that prolong the time scale of preservation of the fermented products. The preservative action of LAB in foods results from the formation of metabolites with antimicrobial activity, e.g., organic acids (lactic, acetic, formic, etc.), hydrogen peroxide (in the presence of oxygen), diacetyl, aldehydes (e.g., β-hydroxy-propionaldehyde) and bacteriocins or bactericidal proteins during lactic fermentation, which make them useful in food bio-preservation.

As the newly identified LAB isolates exhibited high acid and bile salt tolerance, antibiotic resistance, and antibacterial activity, they could be applied as effective and competing probiotic pills for modulating intestinal pathogens and human diseases. Interestingly, pairwise sequence alignment results evidenced a low identity ratio of six isolates (less than 95%) with a high probability to be new species. Further research will be assessed to go through whole genomic sequencing of these isolates, especially the isolate *L. delbrueckii*, ASO 100 that will be subject to complete proteomic analysis to stand for its probiotic determinants, as it revealed the most brilliant probiotic and antibacterial features, along with another in vivo experiment that will be conducted to test the prophylactic and therapeutic ability of this isolate to modulate gut–brain axis microbiota in an Alzheimer’s disease animal model.

## 5. Conclusions

In this study, we screened human breast milk and infant stool samples from Egyptian sources to hunt for innovative Probiotic isolates. Forty-one isolates were submitted to the gene bank database, classified, and identified through physiological and biochemical tests. The representative samples from the different species revealed antibiotic resistance, antibacterial activity, and high probiotic features. Six of our isolates revealed less than 95% Average Nucleotide Identity with other deposited sequences in the database. The isolate *L. delbrueckii*, ASO 100 exhibited the lowest identity ratio with promising probiotic and antibacterial features, casting light on its high probability of being a new probiotic species.

## Figures and Tables

**Table 1:** Morphological properties of lactic acid bacterial isolates.

No	Isolate Code	Isolation Source	Cell Morphology	Gram Stain	Endospore Stain	Catalase Production	Facultatively Anaerobic or Microaerophilic
1	ASO57	Milk	Rods	Positive	Non-endospore	Negative	Positive
2	ASO70	Milk	Rods	Positive	Non-endospore	Negative	Positive
3	ASO62	feces	Cocci	Positive	Non-endospore	Negative	Positive
4	ASO55	Milk	Rods	Positive	Non-endospore	Negative	Positive
5	ASO551	Milk	Rods	Positive	Non-endospore	Negative	Positive
6	ASO53	Milk	Rods	Positive	Non-endospore	Negative	Positive
7	ASO50	Milk	Rods	Positive	Non-endospore	Negative	Positive
8	ASO5	Milk	Rods	Positive	Non-endospore	Negative	Positive
9	ASO46	Milk	Rods	Positive	Non-endospore	Negative	Positive
10	ASO45	Milk	Rods	Positive	Non-endospore	Negative	Positive
11	ASO41	Milk	Cocci	Positive	Non-endospore	Negative	Positive
12	ASO39	Milk	Rods	Positive	Non-endospore	Negative	Positive
13	ASO35	Milk	Rods	Positive	Non-endospore	Negative	Positive
14	ASO290	Milk	Cocci	Positive	Non-endospore	Negative	Positive
15	ASO24	Milk	Rods	Positive	Non-endospore	Negative	Positive
16	ASO23	Milk	Rods	Positive	Non-endospore	Negative	Positive
17	ASO22	Milk	Rods	Positive	Non-endospore	Negative	Positive
18	ASO13	Milk	Rods	Positive	Non-endospore	Negative	Positive
19	ASO102	Milk	Rods	Positive	Non-endospore	Negative	Positive
20	ASO101	Milk	Rods	Positive	Non-endospore	Negative	Positive
21	ASO100	Milk	Rods	Positive	Non-endospore	Negative	Positive
22	ASO103	feces	Cocci	Positive	Non-endospore	Negative	Positive
23	ASO9	Milk	Rods	Positive	Non-endospore	Negative	Positive
24	ASO66	feces	Cocci	Positive	Non-endospore	Negative	Positive
25	ASO65	feces	Cocci	Positive	Non-endospore	Negative	Positive
26	ASO49	Milk	Rods	Positive	Non-endospore	Negative	Positive
27	ASO44	feces	Cocci	Positive	Non-endospore	Negative	Positive
28	ASO421	feces	Cocci	Positive	Non-endospore	Negative	Positive
29	ASO420	feces	Cocci	Positive	Non-endospore	Negative	Positive
30	ASO33	Milk	Rods	Positive	Non-endospore	Negative	Positive
31	ASO32	Milk	Rods	Positive	Non-endospore	Negative	Positive
32	ASO321	Milk	Rods	Positive	Non-endospore	Negative	Positive
33	ASO31	Milk	Rods	Positive	Non-endospore	Negative	Positive
34	ASO292	feces	Cocci	Positive	Non-endospore	Negative	Positive
35	ASO28	feces	Cocci	Positive	Non-endospore	Negative	Positive
36	ASO27	Milk	Rods	Positive	Non-endospore	Negative	Positive
37	ASO26	Milk	Cocci	Positive	Non-endospore	Negative	Positive
38	ASO25	Milk	Rods	Positive	Non-endospore	Negative	Positive
39	ASO20	Milk	Rods	Positive	Non-endospore	Negative	Positive
40	ASO291	feces	Cocci	Positive	Non-endospore	Negative	Positive
41	ASO422	feces	Cocci	Positive	Non-endospore	Negative	Positive

**Table 2:** Physiological and biochemical Characteristics of Cocci lactic acid bacteria.

Isolate No.		ASO62	ASO41	ASO103	ASO66	ASO65	ASO44	ASO421	ASO420	ASO292	ASO28	ASO26	ASO291	ASO422	ASO290
Performed Tests															
Growth at 40 °C		+	+	+	+	+	+	+	+	+	+	+	+	+	+
Growth with 4 % NaCl		+	+	+	+	+	+	+	+	+	+	+	+	+	+
Arginine hydrolysis		+	+	+	+	+	+	+	+	+	+	+	+	+	+
Growth at 10 °C		+	+	+	+	+	+	+	+	+	+	+	+	+	+
Growth at 45 °C		−	−	−	−	−	−	−	−	−	−	−	−	−	−
Growth at pH (9.6)		−	−	−	−	−	−	−	−	−	−	−	−	−	−
Growth at pH (6.8)		+	+	+	+	+	+	+	+	+	+	+	+	+	+
Growth at 6.5% NaCl		+	+	+	+	+	+	+	+	+	+	−	+	+	−
Growth at 4.0% Bile salt		+	+	+	+	+	+	+	+	+	+	+	+	+	+
Acid production from glucose		+	+	+	+	+	+	+	+	+	+	+	+	+	+
Gas production		−	−	−	−	−	−	−	−	−	−	−	−	−	−
Coagulation of milk		+	+	+	+	+	+	+	+	+	+	+	+	+	+
Acid production from	Lactose	+	+	+	+	+	+	+	+	+	+	+	+	+	+
	Mannitol	−	−	−	−	−	−	−	−	−	−	−	−	−	−
	Raffinose	+	+	+	+	+	+	+	+	+	+	+	+	+	+
	Salicin	+	+	+	+	+	+	+	+	+	+	+	+	+	+
	Ribose	−	−	−	−	−	−	−	−	−	−	−	−	−	−
	Trehalose	−	−	−	−	−	−	−	−	−	−	−	−	−	−
	Fructose	+	+	+	+	+	+	+	+	+	+	+	+	+	+
	Sorbitol	−	−	−	−	−	−	−	−	−	−	−	−	−	−
	Glucose	+	+	+	+	+	+	+	+	+	+	+	+	+	+
	Mannose	+	+	+	+	+	+	+	+	+	+	+	+	+	+
	Xylose	−	−	−	−	−	−	−	−	−	−	−	−	−	−

**Table 3:** Physiological and biochemical characteristics of rod lactic acid bacteria.

Isolates No.		ASO 57	ASO 70	ASO 55	ASO 53	ASO 50	ASO 5	ASO 45	ASO 46	ASO 39	ASO 35	ASO 22	ASO 23	ASO 24	ASO 13	ASO9	ASO 49	ASO 31	ASO 32	ASO 33	ASO 20	ASO 25	ASO 27	ASO 101	ASO 102	ASO100
Performed Tests																										
Growth at 15 °C		−	−	−	−	−	−	−	−	−	−	−	−	−	−	−	−	−	−	−	−	−	−	−	−	−
Growth at 45 °C		+	+	+	+	+	+	+	+	+	+	+	+	+	+	+	+	+	+	+	+	+	+	+	+	+
Arginine hydrolysis		+	+	+	+	+	+	+	+	+	+	+	+	+	+	+	+	+	+	+	+	+	+	+	+	+
Gassy production		−	−	−	−	−	−	−	−	−	−	−	−	−	−	−	−	−	−	−	−	−	−	−	−	−
Growth at pH (6.8)		+	+	+	+	+	+	+	+	+	+	+	+	+	+	+	+	+	+	+	+	+	+	+	+	+
Growth at pH (9.6)		+	+	+	+	+	+	+	+	+	+	+	+	+	+	+	+	+	+	+	+	+	+	+	+	+
Growth at 6.5% NaCl		+	+	+	+	+	+	+	+	+	+	+	+	+	+	+	+	+	+	+	+	+	+	+	+	+
Growth with 4.0% bile salt		+	+	+	+	+	+	+	+	+	+	+	+	+	+	+	+	+	+	+	+	+	+	+	+	+
Coagulation of milk		+	+	+	+	+	+	+	+	+	+	+	+	+	+	+	+	+	+	+	+	+	+	+	+	+
Acid production from:	Xylose	−	+	−	+	+	−	+	+	+	+	+	+	+	+	+	+	+	+	+	+	+	+	+	+	−
	Lactose	+	+	+	+	+	+	+	+	+	+	+	+	+	+	+	+	+	+	+	+	+	+	+	+	+
	Galactose	+	+	+	+	+	+	+	+	+	+	+	+	+	+	+	+	+	+	+	+	+	+	+	+	+
	Glucose	+	+	+	+	+	+	+	+	+	+	+	+	+	+	+	+	+	+	+	+	+	+	+	+	+
	Fructose	+	+	+	+	+	+	+	+	+	+	+	+	+	+	+	+	+	+	+	+	+	+	+	+	+
	Maltose	+	+	+	+	+	+	+	+	+	+	+	+	+	+	+	+	+	+	+	+	+	+	+	+	+
	Sucrose	+	+	+	+	+	+	+	+	+	+	+	+	+	+	+	+	+	+	+	+	+	+	+	+	+
	Ribose	−	−	−	−	−	−	−	−	−	−	−	−	−	−	−	−	−	−	−	−	−	−	−	−	−
	Mannitol	−	−	−	−	−	−	−	−	−	−	−	−	−	−	−	−	−	−	−	−	−	−	−	−	−
	Mannose	+	+	+	+	+	+	+	+	+	+	+	+	+	+	+	+	+	+	+	+	+	+	+	+	+
	Raffinose	−	+	−	+	+	+	−	+	+	+	+	+	+	+	+	+	+	+	+	+	+	+	+	+	−
	Rhamnose	+	+	+	+	+	+	+	+	+	+	+	+	+	+	+	+	+	+	+	+	+	+	+	+	+
	Arabinose	+	+	+	+	+	+	+	+	+	+	+	+	+	+	+	+	+	+	+	+	+	+	+	+	+
	Melibiose	+	+	+	+	+	+	+	+	+	+	+	+	+	+	+	+	+	+	+	+	+	+	+	+	+
	Salicin	−	−	−	−	−	−	−	−	−	−	−	−	−	−	−	−	−	−	−	−	−	−	−	−	−
	Sorbitol	−	−	−	−	−	−	−	−	−	−	−	−	−	−	−	−	−	−	−	−	−	−	−	−	−
	Trehalose	−	+	−	+	+	−	+	+	+	+	+	+	+	+	+	+	+	+	+	+	+	+	+	+	−

**Table 4:** The obtained Accession numbers and Identity ratio with the nearest accession in the database.

No	Bacterial Isolate	Accession No.	Identity Ratio	Nearest Accession No.
1	*Lactobacillus paracasei*, ASO 27 Benha	OK033928	98.20%	NR_113823.1
2	*Lactobacillus paracasei*, ASO 31 Benha	OK033924	97.17%	NR_113823.1
3	*Lacticaseibacillus paracasei*, ASO 32 Benha	MZ930465	95.45%	NR_113823.1
4	*Lactobacillus paracasei*, ASO 35 Benha	OK033872	98.94%	NR_113823.1
5	*Lactobacillus paracasei*, ASO 39 Benha	OK033867	97.28%	NR_113823.1
6	*Lactobacillus paracasei*, ASO 45 Benha	OK033489	97.12%	NR_113823.1
7	*Lactobacillus paracasei*, ASO 46 Benha	OK033159	98.81%	NR_113823.1
8	*Lactobacillus paracasei*, ASO 49 Benha	OK033156	97.20%	NR_113823.1
9	*Lactobacillus paracasei*, ASO 53 Benha	OK032621	98.74%	NR_113823.1
10	*Lactobacillus paracasei*, ASO 70 Benha	OK031085	97.85%	NR_113823.1
11	*Lactobacillus paracasei*, ASO 9 Benha	OK035716	96.38%	NR_113823.1
12	*Lactobacillus plantarum*, ASO 33 Benha	OK033922	98.84%	NR_117813.1
13	*Lactobacillus gasseri*, ASO 25 Benha	OK035226	97.14%	NR_075051.2
14	*Lactobacillus plantarum*, ASO 50 Benha	OK033105	96.81%	NR_104573.1
15	*Lactobacillus delbrueckii*, ASO 551 Benha	OK032588	96.64%	NR_113387.1
16	*Lactobacillus delbrueckii*, ASO 57 Benha	OK032511	93.82%	NR_029106.1
17	*Lactobacillus delbrueckii*, ASO 100 Benha	MZ930471	81.39%	NR_029106.1
18	*Lactobacillus delbrueckii*, ASO 55 Benha	MZ930470	96.57%	NR_029106.1
19	*Lactobacillus casei*, ASO 102 Benha	MZ930468	98.66%	NR_113823.1
20	*Lactobacillus paracasei*, ASO 13 Benha	OK020265	97.70%	NR_113823.1
21	*Lactobacillus gasseri*, ASO 22 Benha	OK021660	97.90%	NR_075051.2
22	*Lactobacillus paracasei* ASO 23 Benha	OK021661	97.76%	NR_113823.1
23	*Lactobacillus delbrueckii* ASO 5 Benha	MZ930464	97.66%	NR_029106.1
24	*Lactobacillus paracasei*, ASO 351 Benha	MZ930466	98.62%	NR_113823.1
25	*Lactobacillus paracasei*, ASO 461 Benha	MZ930467	98.57%	NR_113823.1
26	*Lacticaseibacillus paracasei*, ASO 24 Benha	OK035229	97.79%	NR_113823.1
27	*Lacticaseibacillus rhamnosus*, ASO 20 Benha	OK035718	95.70%	NR_113332.1
28	*Lacticaseibacillus paracasei*, ASO 321 Benha	OK033923	95.33%	NR_113823.1
29	*Enterococcus faecium*, ASO 62 Benha	OK032391	97.36%	NR_113904.1
30	*Enterococcus faecium*, ASO 66 Benha	OK032119	94.63%	NR_113904.1
31	*Enterococcus faecium*, ASO 103 Benha	OK030713	95.93%	NR_113904.1
32	*Enterococcus faecium*, ASO 291 Benha	OK030630	95.46%	NR_113904.1
33	*Enterococcus faecium*, ASO 292 Benha	OK030542	93.24%	NR_113904.1
34	*Enterococcus faecium*, ASO 420 Benha	OK021546	96.27%	NR_114742.1
35	*Enterococcus faecium*, ASO 421 Benha	OK021544	93.66%	NR_114742.1
36	*Enterococcus faecalis*, ASO 28 Benha	OK033929	98.14%	NR_113902.1
37	*Enterococcus faecalis*, ASO 44 Benha	OK033573	93.61%	NR_113902.1
38	*Enterococcus faecalis*, ASO 65 Benha	OK032121	95.42%	NR_113902.1
39	*Enterococcus lactis*, ASO 422 Benha	OK020404	98.95%	MT597585.1
40	*Lactococcus lactis*, ASO 26 Benha	OK035225	97.03%	NR_113958.1
41	*Lactococcus lactis*, ASO 290 Benha	OK030692	97.61%	NR_040955.1

**Table 5:** Antibiotic resistance of isolated lactic acid bacteria.

Isolates	Inhibition Zone (mm)				
	Streptomycin (10 μg)	Neomycin (30 μg)	Vancomycin (30 μg)	Tetracycline (30 μg)	Kanamycin (30 μg)
*E. faecalis* ASO44	R *	R	R	R	MS *
*E. faecium* ASO292	R	R	R	R	MS
*L. lactis* ASO26	R	R	R	R	R
*L. delbrueckii* ASO100	MS	R	R	S*	S
*L. casei* ASO53	R	S	R	S	S
*L. plantarum* ASO50	R	S	R	R	R
L. *rhamnosus* ASO20	R	R	MS	R	R
*L. paracasei* ASO32	R	R	R	S	S
*L. gasseri* ASO25	R	R	R	S	R

* Susceptibility is expressed as resistance (R); moderate susceptibility (MS) or susceptibility (S); according to CLSI standards.

**Table 6:** Effect of antimicrobial activity of some lactic acid bacteria on some pathogenic and spoilage bacteria.

Isolates	Inhibition Zone (mm)		
	*B. subtilis*	*E. coli*	*Staph. aureus*
*E. faecalis* ASO44	8.40 ^d^	7.50 ^d^	10.00 ^c^
*E. faecium* ASO292	8.30 ^d^	7.60 ^d^	10.20 ^c^
*L. lactis* ASO26	ND	ND	ND
*L. delbrueckii* ASO100	12.20 ^a^	17.40 ^a^	15.20 ^a^
*L. casei* ASO53	9.70 ^c^	8.00 ^d^	8.50 ^d^
*L. plantarum* ASO50	7.80 ^d^	8.80 ^c^	6.20 ^e^
*L. rhamnosus* ASO20	10.80 ^b^	16.40 ^b^	12.00 ^b^
*L. paracasei* ASO32	7.00 ^e^	5.10 ^e^	11.00 ^bc^
*L. gasseri* ASO25	10.10 ^c^	7.80 ^d^	10.00 ^c^
*SEM*	1.10	0.98	1.21

^a–e^ Estimates with the same letters are not significantly different (*p* > 0.05) among different isolates for the same column. ND stands for Non-Detect.

## Data Availability

Available upon request by corresponding authors.
